# Four Major Factors Contributing to Intrahepatic Stones

**DOI:** 10.1155/2017/7213043

**Published:** 2017-01-09

**Authors:** Xi Ran, Baobing Yin, Baojin Ma

**Affiliations:** ^1^Department of General Surgery, Huashan Hospital, Fudan University, Shanghai, China; ^2^Department of General Surgery, Jing'an District Center Hospital of Shanghai, Fudan University, Shanghai, China

## Abstract

Intrahepatic stone is prevalent in Asian countries; though the incidence declines in recent years, the number of patients is still in a large quantity. Because of multiple complications, high recurrence rates, serious systemic damage, and a lack of extremely effective procedure for the management, it is more important to find out the etiology and pathogenesis of intrahepatic stones to prevent the disease from happening and developing rather than curing. A number of factors contribute to the development of the disease, such as cholestasis, infection, and anatomic abnormity of bile duct and bile metabolic defect. The four factors and possible pathogenesis will be discussed in detail in the review.

## 1. Introduction

Intrahepatic stone is a part of the calculus of bile duct, referring to the stones located proximal to the confluence of the left and/or right hepatic ducts. Intrahepatic stones include brown pigment stones (calcium bilirubin stones), cholesterol stones, and their mixture. Intrahepatic stones are usually accompanied with extrahepatic stones and have properties of multiple complications, high postoperative recurrence rates, and serious systemic damage. The incidence of intrahepatic stone varies among different regions. It is common in East Asia, including China, Japan, and South Korea. The morbidity may reach 18.7% in Taiwan while the average morbidity is only 0.6%–1.3% in western countries [1]. The incidence of intrahepatic stone is declining in recent years [2], but they cannot disappear. The number of patients is still in a large quantity and the problem of refractory and complicated intrahepatic stone is difficult to deal with.

The diagnostic approach, at present, mainly depends on imaging. And the healing therapies now prefer endoscopic treatment approaches to traditional surgical treatment approaches. Endoscopic treatments involve percutaneous transhepatic cholangioscopic lithotripsy (PTCSL), peroral cholangioscopy, and endoscopic retrograde cholangiopancreatography (ERCP). The surgical treatment mainly includes bile duct incision and lithotomy, hepatic resection, reconstruction of bile duct stricture, and liver transplantation. Various treatment strategies have been proposed as mentioned above since there is no extremely effective procedure for the management of intrahepatic stone. So, it is more important to find out the etiology and pathogenesis of intrahepatic stones to prevent the disease from happening and developing rather than curing. Although the pathogenesis of intrahepatic stones has not been disclosed clear so far, it is still reported to be related with the environment, nutritional status, bile duct infection, cholestasis, parasites, the variation of bile duct, bile metabolic defect, and so forth. These factors may cooperate to induce intrahepatic stones. Among these factors, cholestasis, infection, and anatomic abnormity of bile duct and bile metabolic defect occupy the most important positions.

## 2. Cholestasis

Bile is produced by liver cells and finally excreted into duodenum to help in digestion and absorption. During the period of nondigestion, most of the bile is stored in gallbladder. Food is the natural stimulant which causes the secretion and excretion of bile. High-protein food is the biggest stimulating effect of bile, then comes high-fat food or mixed food, and the carbohydrate food is the smallest. In addition, the coordination of gallbladder and Sphincter of Oddi (SO) contraction and relaxation is also playing important role in the process of bile excretion. Bile salts are the major solutes, which form the main permeability power of the bile flow. Any factor which affects bile production and excretion can cause cholestasis.

Cholestasis is essential for the formation of intrahepatic stone, both pigment stones and cholesterol stones. It can be caused by viruses, bacteria, parasites, drugs or toxins, stones, tumors, self-immunity, genetic metabolic defects, and obstruction. As a result, bile acid is accumulated in liver and blood and may bring injury. Sometimes, there is no mechanical obstruction in the distal common bile duct. However, the functional obstruction which is formed by distal common bile duct edema and SO spasm caused by cholangitis cannot be excluded. Above the obstruction, the biliary duct pressure increases, the bile flow slows down, and then cholestasis comes into being. East Asians prefer low-fat and low-protein diets, thus having negative effect on bile empty and leading to cholestasis. Besides, low-protein diets may reduce the level of *β*-glucuronidase inhibitor in bile contributing to the formation of pigment stones. They also cause malnutrition which results in decreased damage resistance, increasing the incidence of stricture of bile duct and bacterial infection. Moreover, since gallbladder and SO play important roles in bile excretion, patients with cholecystectomy or dysfunction of SO may also be confronted with the disorder of biliary motility and bile excretion, leading to cholestasis. Cholestasis provides time and place for the bile components deposited and then form shaped stones, while cholestasis also means toxic bile acid accumulated to cause biliary walls injury and inflammation [3]. And these then provide a condition for ascending infection. This is a vicious cycle. Once biliary sludge formed, bile duct obstruction is aggravated.

## 3. Infection

Intrahepatic bile duct infection includes bacteria and parasites. Infection is perhaps the prime cause for pigment stones. Almost in all the patients with intrahepatic stone, bacteria can be detected on bile culture. Clemente et al. [4] isolated 133 microorganisms from bile of 73 patients with intrahepatic stone, among which* Escherichia coli* (*E. coli*) is the most common one. Apart from* Enterobacter*, anaerobic bacteria can also be detected. The possible infective ways may include ascending infection via SO, hematogenous dissemination via portal system, and spreading through lymphatic system. In the three ways, ascending infection via SO is the most important. Reflux cholangitis and stones have been widely recognized. Once SO stops working properly, the function of one-way valve disappeared and duodenal fluid refluxes into bile duct, causing the damage to the bile duct mucous membrane with bacterial invasion and then reflux cholangitis. And these in turn promote the recurrence of stones.

The pathogenesis of bacterial infection has been explained relatively clearly. The bacteria in bile duct produce *β*-glucuronidase which can hydrolyze conjugated bilirubin to unconjugated bilirubin. Then, unconjugated bilirubin and ionized calcium will combine together and form calcium bilirubinate deposited. Besides, bacteria also produce phospholipase A1, turning phosphatidylcholine to free fatty acids and lysophosphatidylcholine, consequently accelerating the deposition of fatty acid calcium and secretion of mucin from bile duct epithelium promoting the formation of stones. Moreover, the endotoxins produced by bacteria activate the liver cells, bile duct epithelium cells, and white blood cells in bile to release the endogenous *β*-glucuronidase contributing to the form of stones. In addition, bacterial lipopolysaccharide (LPS) increases mucin 5AC (MUC5AC) by tumor necrosis factor-*α* converting enzyme (TACE)/transforming growth factor-*α* (TGF-*α*)/growth factor receptor (EGFR) pathway in intrahepatic biliary epithelial cells also involved in the formulation and development of intrahepatic stone [5]. Meanwhile, bacterial infection will aggravate cholestasis and cholangitic stenosis to form a vicious circle, continuously stimulating the formation and growth of stones [6]. Lately,* Helicobacter pylori* (*H. pylori*) has been found to be related with the occurrence of intrahepatic stone as well [7].* H. pylori* was believed to promote the proliferation of bile duct epithelium which played an important role in the process that intrahepatic stone eventually developed into cholangiocarcinoma.

Parasites, such as* Ascaris*, liver fluke (including* Clonorchis sinensis*, and* Opisthorchis viverrini*), and* Schistosoma*, account for intrahepatic stone as well. They can not only damage the epithelium of bile duct to cause inflammation but also form the nucleus of stones with their body. For example, the pathogenic mechanisms of* Ascaris* may include* Ascaris* excreting various types of polypeptides producing allergic manifestations which lead to biliary walls damage and bile duct inflammatory. The dead worms and the eggs form the nucleus of stones, and the worms block bile duct causing cholestasis and bacterial infection [8]. Besides, these worms have high glucuronidase activity, which deconjugates bilirubin and forms pigment stones.

Similarly, an association between liver fluke infection and intrahepatic stone is well recognized. As one kind of liver fluke,* Clonorchis sinensis* (*C. sinensis*) infection is believed to be associated with intrahepatic stones, especially pigment stones. It is shown that the adult flukes are predominantly present in intrahepatic bile ducts and intrahepatic stones are one of the main complications of* C. sinensis* infection secondary to mechanical injury and obstruction of the bile duct epithelium by flukes and chronic inflammation [9].* C. sinensis* eggs were also found inside as nucleus of stones. The uneven inherent texture of* C. sinensis* eggs is easy for bile components to adhere to its surface. Moreover, under the effects of* C. sinensis*, goblet cell metaplasia and mucin secretion of bile duct epithelium occurred, making eggs surrounded by particles around [9]. Therefore, the bile rich in mucin and flukes induces a favorable environment for secondary bacterial infection through ascending infection [10]. Thus,* C. sinensis* as well as intrahepatic stones is found frequently in patients with recurrent pyogenic cholangitis [11]. The same results have been obtained in the experiments of sheep [12]. However, apart from pigment stones, cholesterol stones were found in nearly half of the cases.* Opisthorchis viverrini (O. viverrini)* have been observed in the stones of infected or previously treated individuals, just as* C. sinensis* eggs were found in the stones of those with Chinese liver fluke infection [13]. In addition, heavy* O. viverrini* infection can induce poor emptying of the gallbladder, resulting in cholestasis, ascending cholangitis, and accelerated stone formation. Besides, repeated infections and antifluke medication treatments may produce dead worms contributing to the formation of stones [14].

However, the infection of parasites may not be the leading cause of intrahepatic stone. On the one hand, the epidemic areas of parasites are different from the epidemic areas of intrahepatic stone in Taiwan and we can rarely detect parasites and their eggs in stones; on the other hand, the incidence of intrahepatic stone in Japan is higher than western countries though intestinal parasites have been eradicated there [2].

## 4. Anatomic Abnormality of Bile Duct

Intrahepatic stones are easy to occur in intrahepatic bile duct with anatomic variation and poor bile evacuation. For example, the left hepatic duct is slender and meets common hepatic duct at nearly a right angel, and there is often a corner when the right posterior segmental duct meets the right hepatic duct. The two cases mentioned above lead to bile excretion disorder and cholestasis. So the stones of right posterior lobe type and left lobe type are the most common ones. Apart from normal physiological and anatomical influence, the congenital or acquired anatomic abnormity, deformity, or disease, such as anastomotic stricture, congenital choledochal cyst, and Caroli's disease, can also increase the morbidity of intrahepatic stones. Quite a few studies have shown that the recurrence rate of patients with strictures after operation is significantly higher than that without strictures [15–17]. A multivariate analysis showed that stricture was an independent risk factor of residual stones, cholangitis, and stone recurrence after treatment [15]. And it has been shown that patients with congenital choledochal cyst have low incidence of intrahepatic stone, while the incidence becomes higher after the resection of dilated bile duct and reconstruction of biliary tract. When bile flows through biliary duct with strictures, the bile generates circumfluence and vortex above and below the stricture, providing additional power for small particles gathering.

**Figure 1:**
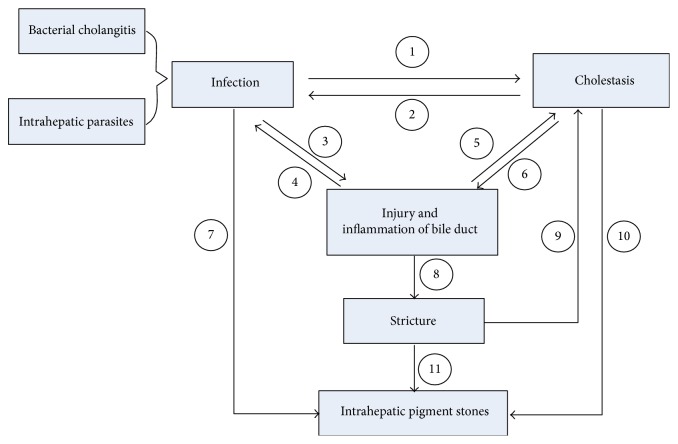
Causes of intrahepatic pigments stones and their relationships. Infection, cholestasis, and stricture are the three main causes of intrahepatic pigment stones. ① Bacterial infection may influence the produce and transport of the bile and the worms or eggs of the parasites may lead to obstruction, resulting in cholestasis. ② It is easier for bacteria to invade when cholestasis. ③ Bacteria or worms as well as their secretions are easy to induce the injury and inflammation of bile duct. ④ Bacterial cholangitis is inclined to occur after the loss of protective effects of normal bile duct. ⑤  ⑨ The pressure inside the bile duct changes when there is injury, inflammation, or stricture of bile duct, leading to cholestasis. ⑥ Cholestasis means toxic bile acid accumulated to cause biliary walls injury and inflammation. ⑦ Bacteria produce relevant substances that may cause changes of the components in bile and finally deposit. ⑧ Prolonged inflammation may cause fibrosis of biliary walls, resulting in cholangitic stenosis. ⑩ Cholestasis provides time and place for the bile components deposited and then forms shaped stones. ⑪ The bile generates circumfluence and vortex above and below the stricture, providing additional power for small particles gathering.

The common pathological changes in intrahepatic stone are stones, strictures, dilation, chronic proliferative cholangitis, cholangiocarcinoma, hepatic parenchymal fibrosis, and hepatic atrophy [18]. Stricture is the major pathological change and the common reason for stone recurrence and surgical treatment failure [1]. Except for these factors above, stricture caused by ischemia and varices of bile duct are relevant to intrahepatic stone as well [19]. When biliary dilatation or stricture occurred, cholestasis occurred subsequently and caused infection. Stones, inflammation, and strictures influence each other and are reciprocal causation, promoting the formation and development of intrahepatic stone concurrently (Figure 1).

## 5. Bile Metabolic Defect

The most important reason for cholesterol stones is the imbalance of bile components associated with bile metabolic defect. In Japan, since cases of primary intrahepatic cholesterol stones were emerging, a series component analysis of stones have been conducted and these demonstrated that cholesterol level in intrahepatic cholesterol stones was higher than extrahepatic ones. The mechanisms may be related to the increase of cholesterol, the reduction of bile acid, and phospholipid secretion defects. ABCB4 codes for multidrug resistance protein 3 (MDR3), which transports phosphatidylcholine from the inside to the outside of liver cell canalicular membrane. Studies have covered that the variants of the ABCB4 gene have been identified to be associated with intrahepatic stone by influencing the content of phosphatidylcholine in bile [20]. ABCB11 codes for the bile salt export pump (BSEP), which mediates the secretion of bile salts from the liver cells into the bile duct. Simultaneously, the variants of the ABCB11 gene have also been shown to be related to intrahepatic stone through decreasing the secretion of bile acid [20].

Besides, the changes of bile components and the oversecretion of mucins caused by the activation of arachidonic acid lead to cholestasis and promote crystallization of cholesterol, resulting in cholesterol stones or stones rich in cholesterol. The level of mucins including MUC1, MUC2, MUC3, MUC5AC, and MUC6 in bile duct with intrahepatic stone is obviously higher in bile duct without intrahepatic stone. These mucins have properties of poor liquidity and may form gel in bile duct. Among these mucins, MUC2 and MUC5AC are reported to be involved in chronic proliferative cholangitis and intrahepatic stone after acute cholangitis. For instance, PGE2 induces MUC2 and MUC5AC expression via EP4-p38MAPK signaling in intrahepatic biliary epithelial cells, contributing to the formulation and development of intrahepatic stone [21]. Furthermore, it is demonstrated that nuclear receptor, such as farnesoid X receptor (FXR), has an effect in the development of intrahepatic stone as well. In the experiment, FXR-knockout mice have elevated serum cholesterol and triglyceride levels, resulting in elevation of cholesterol in bile and then making a contribution to the development of stones [22].

## 6. Summary

Intrahepatic stone is a prevalent disease in East Asian countries. Finding out a highly effective treatment strategy of the disease is always the focus of the considerable concern. However, exploring the etiology and pathogenesis of intrahepatic stone and preventing the disease from happening and developing may be potentially more significant. Congenital and acquired risk factors predisposing to the disease have been detected. Four chief factors are cholestasis, infection, anatomic abnormity of bile duct, and bile metabolic defect. Lacking highly effective procedure for the management though, more strategies for the prevention and treatment will be formulated with the further study of the etiology and pathogenesis of intrahepatic stones.
